# 2-Isobutyl-6-(4-meth­oxy­phen­yl)imidazo[2,1-*b*][1,3,4]thia­diazole

**DOI:** 10.1107/S1600536810053225

**Published:** 2011-01-08

**Authors:** Hoong-Kun Fun, Chin Sing Yeap, D. Jagadeesh Prasad, Prakash Anil Castelino, V. V. Anitha

**Affiliations:** a X-ray Crystallography Unit, School of Physics, Universiti Sains Malaysia, 11800 USM, Penang, Malaysia; b Department of Chemistry, Mangalore University, Mangalore, Karnataka, India; c St. Philomena’s College, Puttur, Dakshina Kannada, Karnataka, India

## Abstract

In the title compound, C_15_H_17_N_3_OS, the dihedral angle between the statistically planar imidazo[2,1-*b*][1,3,4]thia­dia­zole fused-ring system (r.m.s. deviation = 0.002 Å) and the methyoxbenzene ring is 4.52 (6)°. In the crystal, mol­ecules are arranged into columns and stacked down the *a* axis. The crystal structure is stabilized by weak C—H⋯π and π–π inter­actions [centroid–centroid separations = 3.6053 (8) and 3.7088 (7) Å].

## Related literature

For a related structure and background references to imidazo[2,1-*b*]-1,3,4-thia­diazole derivatives, see: Fun *et al.* (2011 ▶).
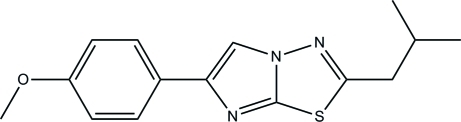


## Experimental

### 

#### Crystal data

C_15_H_17_N_3_OS
*M* *_r_* = 287.38
Triclinic, 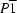

*a* = 5.7139 (1) Å
*b* = 10.1795 (1) Å
*c* = 12.9689 (2) Å
α = 85.174 (1)°
β = 85.164 (1)°
γ = 80.690 (1)°
*V* = 739.84 (2) Å^3^
*Z* = 2
Mo *K*α radiation
μ = 0.22 mm^−1^
*T* = 296 K
0.43 × 0.31 × 0.17 mm

#### Data collection

Bruker SMART APEXII CCD diffractometer
Absorption correction: multi-scan (*SADABS*; Bruker, 2009 ▶) *T*_min_ = 0.911, *T*_max_ = 0.964
23503 measured reflections
6213 independent reflections
3805 reflections with *I* > 2σ(*I*)
*R*_int_ = 0.028

#### Refinement

*R*[*F* ^2^ > 2σ(*F* ^2^)] = 0.053
*wR*(*F* ^2^) = 0.163
*S* = 1.03
6213 reflections
181 parameters
H-atom parameters constrained
Δρ_max_ = 0.30 e Å^−3^
Δρ_min_ = −0.28 e Å^−3^

### 

Data collection: *APEX2* (Bruker, 2009 ▶); cell refinement: *SAINT* (Bruker, 2009 ▶); data reduction: *SAINT*; program(s) used to solve structure: *SHELXTL* (Sheldrick, 2008 ▶); program(s) used to refine structure: *SHELXTL*; molecular graphics: *SHELXTL*; software used to prepare material for publication: *SHELXTL* and *PLATON* (Spek, 2009 ▶).

## Supplementary Material

Crystal structure: contains datablocks global, I. DOI: 10.1107/S1600536810053225/hb5778sup1.cif

Structure factors: contains datablocks I. DOI: 10.1107/S1600536810053225/hb5778Isup2.hkl

Additional supplementary materials:  crystallographic information; 3D view; checkCIF report

## Figures and Tables

**Table 1:** Hydrogen-bond geometry (Å, °) *Cg*3 is the centroid of the C1–C6 benzene ring.

*D*—H⋯*A*	*D*—H	H⋯*A*	*D*⋯*A*	*D*—H⋯*A*
C11—H11*A*⋯*Cg*3^i^	0.97	2.60	3.5063 (16)	155

Symmetry code: (i) 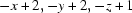.

**Fig. 1.**
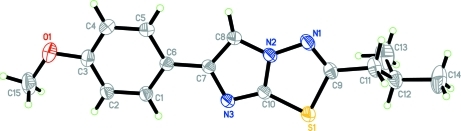
The molecular structure of (I) with 30% probability ellipsoids for non-H atoms.

**Fig. 2.**
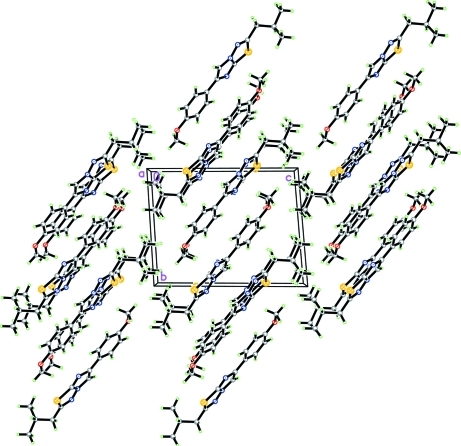
The crystal packing of (I), viewed down the a axis, showing molecules stacked down a axis.

## supplementary crystallographic information

### Comment

As part of our ongoing synthetic and structural studies of imidazo[2,1-*b*]-1,3,4-thiadiazole derivatives (Fun *et al.*, 2011), we now report the structure of the title compound, (I).

The mean plane through the imidazo[2,1-*b*]-1,3,4-thiadiazole ring and the methoxyphenyl moiety is essentially planar with the maximum deviation of 0.045 Å for atom C2 (Fig. 1). The isobutyl is twisted away from this mean plane with torsion angles of C9–C11–C12–C13 = 64.3 (2)° and C9–C11–C12–C14 = -172.81 (17)°. In the crystal structure, the molecules are arranged into columns and stacked down *a* axis (Fig. 2). The molecules are stabilized by the weak *Cg*1···*Cg*2^i^ = 3.7088 (7) Å, *Cg*2···*Cg*2^i^ = 3.6053 (8) Å and C11–H11A···*Cg*3^i^ interactions [*Cg*1, *Cg*2 and *Cg*3 are centroids of S1/C9/N1/N2/C10, N2/C8/C7/N3/C10 and C–C6 ring respectively; (i) 2 - *x*, 2 - *y*, 1 - *z*].

### Experimental

5-Isobutyl-1,3,4-thiadiazol-2-amine (1 molar equivalent) and 4-methoxyphenacylbromide (1 molar equivalent) are refluxed with ethanol for 4 h. The solvent was then distilled and the reaction mass was poured onto the crushed ice. The resulting solid that separated out was filtered and dried. The compound was re-crystallized using ethanol and DMF mixture to yield colourless blocks of (I). M.P.: 118–122°C.

### Refinement

All hydrogen atoms were positioned geometrically [C–H = 0.93–0.98 Å] and refined using a riding model, with *U*_iso_(H) = 1.2 or 1.5*U*_eq_(C).

### Crystal data

**Table:**

C_15_H_17_N_3_OS	*Z* = 2
*M**_r_* = 287.38	*F*(000) = 304
Triclinic, *P*1	*D*_x_ = 1.290 Mg m^−^^3^
Hall symbol: -P 1	Mo *K*α radiation, λ = 0.71073 Å
*a* = 5.7139 (1) Å	Cell parameters from 6150 reflections
*b* = 10.1795 (1) Å	θ = 2.5–30.1°
*c* = 12.9689 (2) Å	µ = 0.22 mm^−^^1^
α = 85.174 (1)°	*T* = 296 K
β = 85.164 (1)°	Block, colourless
γ = 80.690 (1)°	0.43 × 0.31 × 0.17 mm
*V* = 739.84 (2) Å^3^	

### Data collection

**Table:**

Bruker SMART APEXII CCD diffractometer	6213 independent reflections
Radiation source: fine-focus sealed tube	3805 reflections with *I* > 2σ(*I*)
graphite	*R*_int_ = 0.028
φ and ω scans	θ_max_ = 34.5°, θ_min_ = 1.6°
Absorption correction: multi-scan (*SADABS*; Bruker, 2009)	*h* = −9→9
*T*_min_ = 0.911, *T*_max_ = 0.964	*k* = −16→16
23503 measured reflections	*l* = −20→20

### Refinement

**Table:**

Refinement on *F*^2^	Primary atom site location: structure-invariant direct methods
Least-squares matrix: full	Secondary atom site location: difference Fourier map
*R*[*F*^2^ > 2σ(*F*^2^)] = 0.053	Hydrogen site location: inferred from neighbouring sites
*wR*(*F*^2^) = 0.163	H-atom parameters constrained
*S* = 1.03	*w* = 1/[σ^2^(*F*_o_^2^) + (0.0765*P*)^2^ + 0.0748*P*] where *P* = (*F*_o_^2^ + 2*F*_c_^2^)/3
6213 reflections	(Δ/σ)_max_ < 0.001
181 parameters	Δρ_max_ = 0.30 e Å^−^^3^
0 restraints	Δρ_min_ = −0.28 e Å^−^^3^

### Special details

**Table:**

Geometry. All e.s.d.'s (except the e.s.d. in the dihedral angle between two l.s. planes) are estimated using the full covariance matrix. The cell e.s.d.'s are taken into account individually in the estimation of e.s.d.'s in distances, angles and torsion angles; correlations between e.s.d.'s in cell parameters are only used when they are defined by crystal symmetry. An approximate (isotropic) treatment of cell e.s.d.'s is used for estimating e.s.d.'s involving l.s. planes.
Refinement. Refinement of *F*^2^ against ALL reflections. The weighted *R*-factor *wR* and goodness of fit *S* are based on *F*^2^, conventional *R*-factors *R* are based on *F*, with *F* set to zero for negative *F*^2^. The threshold expression of *F*^2^ > σ(*F*^2^) is used only for calculating *R*-factors(gt) *etc*. and is not relevant to the choice of reflections for refinement. *R*-factors based on *F*^2^ are statistically about twice as large as those based on *F*, and *R*- factors based on ALL data will be even larger.

### Fractional atomic coordinates and isotropic or equivalent isotropic displacement parameters (Å^2^)

**Table:**

	*x*	*y*	*z*	*U*_iso_*/*U*_eq_	
S1	1.14439 (6)	1.02701 (4)	0.26563 (3)	0.05911 (14)	
O1	1.1085 (2)	0.33486 (12)	0.78317 (9)	0.0741 (3)	
N1	0.7031 (2)	1.05243 (12)	0.33719 (9)	0.0523 (3)	
N2	0.82991 (18)	0.94641 (11)	0.39100 (8)	0.0448 (2)	
N3	1.16801 (18)	0.81311 (11)	0.42311 (8)	0.0470 (2)	
C1	1.2380 (2)	0.58244 (13)	0.57132 (10)	0.0459 (3)	
H1A	1.3637	0.6030	0.5258	0.055*	
C2	1.2769 (3)	0.47212 (13)	0.64264 (10)	0.0498 (3)	
H2A	1.4261	0.4199	0.6442	0.060*	
C3	1.0917 (3)	0.44125 (14)	0.71078 (10)	0.0515 (3)	
C4	0.8704 (3)	0.52078 (17)	0.70788 (12)	0.0620 (4)	
H4A	0.7456	0.5008	0.7542	0.074*	
C5	0.8345 (2)	0.62946 (16)	0.63658 (12)	0.0541 (3)	
H5A	0.6852	0.6817	0.6354	0.065*	
C6	1.0186 (2)	0.66223 (12)	0.56621 (9)	0.0405 (2)	
C7	0.9808 (2)	0.77580 (12)	0.48936 (9)	0.0397 (2)	
C8	0.7710 (2)	0.85747 (13)	0.47028 (10)	0.0497 (3)	
H8A	0.6217	0.8533	0.5038	0.060*	
C9	0.8461 (2)	1.10357 (13)	0.26920 (10)	0.0473 (3)	
C10	1.0665 (2)	0.91576 (13)	0.36590 (9)	0.0441 (3)	
C11	0.7678 (3)	1.22647 (14)	0.20133 (11)	0.0571 (4)	
H11A	0.8370	1.2992	0.2240	0.069*	
H11B	0.5965	1.2493	0.2123	0.069*	
C12	0.8305 (3)	1.21810 (16)	0.08633 (11)	0.0642 (4)	
H12A	1.0026	1.1905	0.0754	0.077*	
C13	0.7083 (6)	1.1178 (2)	0.04179 (17)	0.1097 (9)	
H13A	0.7482	1.0322	0.0783	0.165*	
H13B	0.7594	1.1119	−0.0303	0.165*	
H13C	0.5393	1.1455	0.0489	0.165*	
C14	0.7663 (6)	1.3557 (2)	0.03141 (17)	0.1117 (9)	
H14A	0.8461	1.4183	0.0607	0.167*	
H14B	0.5975	1.3836	0.0400	0.167*	
H14C	0.8148	1.3522	−0.0411	0.167*	
C15	1.3354 (4)	0.25718 (19)	0.79377 (16)	0.0840 (6)	
H15A	1.3233	0.1863	0.8467	0.126*	
H15B	1.4436	0.3125	0.8127	0.126*	
H15C	1.3931	0.2200	0.7291	0.126*	

### Atomic displacement parameters (Å^2^)

**Table:**

	*U*^11^	*U*^22^	*U*^33^	*U*^12^	*U*^13^	*U*^23^
S1	0.04595 (19)	0.0694 (3)	0.0549 (2)	−0.00092 (16)	0.00022 (14)	0.01745 (17)
O1	0.0866 (8)	0.0673 (7)	0.0650 (7)	−0.0162 (6)	−0.0051 (6)	0.0229 (5)
N1	0.0439 (6)	0.0567 (7)	0.0504 (6)	0.0074 (5)	−0.0069 (5)	0.0047 (5)
N2	0.0364 (5)	0.0504 (6)	0.0438 (5)	0.0022 (4)	−0.0044 (4)	0.0022 (4)
N3	0.0361 (5)	0.0551 (6)	0.0462 (6)	−0.0009 (4)	−0.0031 (4)	0.0060 (4)
C1	0.0453 (6)	0.0489 (7)	0.0394 (6)	0.0004 (5)	0.0018 (5)	0.0001 (5)
C2	0.0532 (7)	0.0483 (7)	0.0440 (6)	0.0019 (5)	−0.0021 (5)	−0.0004 (5)
C3	0.0644 (8)	0.0468 (7)	0.0441 (6)	−0.0126 (6)	−0.0077 (6)	0.0034 (5)
C4	0.0531 (8)	0.0731 (10)	0.0584 (8)	−0.0177 (7)	0.0027 (6)	0.0119 (7)
C5	0.0394 (6)	0.0654 (8)	0.0546 (7)	−0.0063 (6)	0.0001 (5)	0.0060 (6)
C6	0.0395 (6)	0.0448 (6)	0.0374 (5)	−0.0054 (5)	−0.0040 (4)	−0.0050 (4)
C7	0.0372 (5)	0.0445 (6)	0.0365 (5)	−0.0023 (4)	−0.0035 (4)	−0.0050 (4)
C8	0.0375 (6)	0.0565 (8)	0.0505 (7)	−0.0008 (5)	0.0019 (5)	0.0051 (6)
C9	0.0514 (7)	0.0489 (7)	0.0394 (6)	0.0027 (5)	−0.0096 (5)	−0.0042 (5)
C10	0.0364 (6)	0.0528 (7)	0.0409 (6)	−0.0022 (5)	−0.0038 (4)	0.0010 (5)
C11	0.0711 (9)	0.0495 (7)	0.0465 (7)	0.0050 (6)	−0.0117 (6)	0.0006 (5)
C12	0.0702 (10)	0.0673 (9)	0.0486 (8)	0.0017 (8)	−0.0023 (7)	0.0078 (7)
C13	0.175 (3)	0.0923 (15)	0.0672 (12)	−0.0145 (17)	−0.0461 (15)	−0.0119 (11)
C14	0.164 (3)	0.0872 (14)	0.0717 (13)	−0.0059 (16)	−0.0039 (14)	0.0319 (11)
C15	0.1056 (16)	0.0615 (10)	0.0816 (12)	−0.0074 (10)	−0.0270 (11)	0.0232 (9)

### Geometric parameters (Å, °)

**Table:**

S1—C10	1.7290 (13)	C6—C7	1.4622 (16)
S1—C9	1.7545 (14)	C7—C8	1.3722 (17)
O1—C3	1.3690 (16)	C8—H8A	0.9300
O1—C15	1.416 (2)	C9—C11	1.4966 (18)
N1—C9	1.2864 (19)	C11—C12	1.510 (2)
N1—N2	1.3721 (14)	C11—H11A	0.9700
N2—C10	1.3554 (16)	C11—H11B	0.9700
N2—C8	1.3689 (16)	C12—C13	1.503 (3)
N3—C10	1.3133 (15)	C12—C14	1.521 (2)
N3—C7	1.3957 (16)	C12—H12A	0.9800
C1—C6	1.3825 (17)	C13—H13A	0.9600
C1—C2	1.3947 (17)	C13—H13B	0.9600
C1—H1A	0.9300	C13—H13C	0.9600
C2—C3	1.379 (2)	C14—H14A	0.9600
C2—H2A	0.9300	C14—H14B	0.9600
C3—C4	1.388 (2)	C14—H14C	0.9600
C4—C5	1.381 (2)	C15—H15A	0.9600
C4—H4A	0.9300	C15—H15B	0.9600
C5—C6	1.3967 (18)	C15—H15C	0.9600
C5—H5A	0.9300		
			
C10—S1—C9	88.40 (6)	N3—C10—N2	112.76 (11)
C3—O1—C15	117.66 (14)	N3—C10—S1	138.85 (10)
C9—N1—N2	108.55 (11)	N2—C10—S1	108.39 (9)
C10—N2—C8	107.69 (10)	C9—C11—C12	116.36 (12)
C10—N2—N1	118.51 (11)	C9—C11—H11A	108.2
C8—N2—N1	133.79 (11)	C12—C11—H11A	108.2
C10—N3—C7	103.73 (10)	C9—C11—H11B	108.2
C6—C1—C2	122.09 (12)	C12—C11—H11B	108.2
C6—C1—H1A	119.0	H11A—C11—H11B	107.4
C2—C1—H1A	119.0	C13—C12—C11	112.03 (16)
C3—C2—C1	119.33 (13)	C13—C12—C14	110.67 (17)
C3—C2—H2A	120.3	C11—C12—C14	109.10 (15)
C1—C2—H2A	120.3	C13—C12—H12A	108.3
O1—C3—C2	124.39 (14)	C11—C12—H12A	108.3
O1—C3—C4	115.99 (14)	C14—C12—H12A	108.3
C2—C3—C4	119.62 (13)	C12—C13—H13A	109.5
C5—C4—C3	120.36 (14)	C12—C13—H13B	109.5
C5—C4—H4A	119.8	H13A—C13—H13B	109.5
C3—C4—H4A	119.8	C12—C13—H13C	109.5
C4—C5—C6	121.14 (14)	H13A—C13—H13C	109.5
C4—C5—H5A	119.4	H13B—C13—H13C	109.5
C6—C5—H5A	119.4	C12—C14—H14A	109.5
C1—C6—C5	117.46 (12)	C12—C14—H14B	109.5
C1—C6—C7	121.12 (11)	H14A—C14—H14B	109.5
C5—C6—C7	121.42 (11)	C12—C14—H14C	109.5
C8—C7—N3	111.03 (11)	H14A—C14—H14C	109.5
C8—C7—C6	127.62 (11)	H14B—C14—H14C	109.5
N3—C7—C6	121.35 (10)	O1—C15—H15A	109.5
N2—C8—C7	104.79 (11)	O1—C15—H15B	109.5
N2—C8—H8A	127.6	H15A—C15—H15B	109.5
C7—C8—H8A	127.6	O1—C15—H15C	109.5
N1—C9—C11	122.38 (13)	H15A—C15—H15C	109.5
N1—C9—S1	116.15 (10)	H15B—C15—H15C	109.5
C11—C9—S1	121.36 (11)		
			
C9—N1—N2—C10	0.23 (17)	C10—N2—C8—C7	0.06 (15)
C9—N1—N2—C8	179.94 (14)	N1—N2—C8—C7	−179.67 (13)
C6—C1—C2—C3	−0.3 (2)	N3—C7—C8—N2	0.03 (15)
C15—O1—C3—C2	4.8 (2)	C6—C7—C8—N2	−179.34 (11)
C15—O1—C3—C4	−175.43 (15)	N2—N1—C9—C11	−176.23 (11)
C1—C2—C3—O1	179.30 (13)	N2—N1—C9—S1	−0.04 (15)
C1—C2—C3—C4	−0.4 (2)	C10—S1—C9—N1	−0.10 (12)
O1—C3—C4—C5	−179.11 (14)	C10—S1—C9—C11	176.13 (11)
C2—C3—C4—C5	0.7 (2)	C7—N3—C10—N2	0.14 (15)
C3—C4—C5—C6	−0.2 (2)	C7—N3—C10—S1	−179.92 (13)
C2—C1—C6—C5	0.7 (2)	C8—N2—C10—N3	−0.13 (16)
C2—C1—C6—C7	−178.73 (12)	N1—N2—C10—N3	179.65 (11)
C4—C5—C6—C1	−0.5 (2)	C8—N2—C10—S1	179.92 (9)
C4—C5—C6—C7	178.95 (13)	N1—N2—C10—S1	−0.31 (15)
C10—N3—C7—C8	−0.10 (14)	C9—S1—C10—N3	−179.73 (16)
C10—N3—C7—C6	179.31 (11)	C9—S1—C10—N2	0.21 (10)
C1—C6—C7—C8	175.00 (13)	N1—C9—C11—C12	−129.32 (16)
C5—C6—C7—C8	−4.5 (2)	S1—C9—C11—C12	54.69 (18)
C1—C6—C7—N3	−4.31 (18)	C9—C11—C12—C13	64.3 (2)
C5—C6—C7—N3	176.24 (12)	C9—C11—C12—C14	−172.81 (17)

### Hydrogen-bond geometry (Å, °)

**Table:**

Cg3 is the centroid of the C1–C6 benzene ring.

**Table:**

*D*—H···*A*	*D*—H	H···*A*	*D*···*A*	*D*—H···*A*
C11—H11A···Cg3^i^	0.97	2.60	3.5063 (16)	155

Symmetry codes: (i) −*x*+2, −*y*+2, −*z*+1.
